# Transcriptomic Analysis of *Mucor irregularis* Containing a Negative Single-Stranded RNA Mycovirus

**DOI:** 10.1128/MRA.00503-19

**Published:** 2019-07-25

**Authors:** Rafael R. Barata, João L. S. G. Vianez-Júnior, Márcio R. T. Nunes

**Affiliations:** a Center for Technological Innovations, Evandro Chagas Institute, Ananindeua, Pará, Brazil; University of California, Riverside

## Abstract

The fungus Mucor irregularis is a causative agent of mucormycosis. The transcriptome analysis of the isolated M. irregularis strain C3B revealed the presence of an RNA polymerase domain of a negative-polarity RNA virus. In this work, we describe the gene ontology-based annotation of the *Mucor irregularis* transcriptome, which includes a putative RNA mycovirus.

## ANNOUNCEMENT

The fungus Mucor irregularis is a causative agent of mucormycosis, especially in people with an impaired immune system; however, it can also affect immunocompetent hosts, causing primary cutaneous mycosis (1, 2).

*Mucor irregularis* isolate C3B was obtained from a soil sample from the environmental protection area Combu Island (1°29′45.0″S, 48°27′38.6″W), a region of the Amazon forest, located south of the city of Belém, Pará State, Brazil. The soil sample was collected, serially diluted to 1 mg/ml, and inoculated in potato dextrose agar.

For total RNA extraction, the fungal mycelium was transferred to a microtube containing Tris-EDTA buffer and 5-mm beads. Then, thermal lysis was carried out by three successive immersion cycles of the microtube in water at 100°C for 1 min and liquid nitrogen for 30 s. Subsequently, the tube was vigorously shaken in the TissueLyser II (Qiagen) for mechanical lysis. Total RNA purification was performed using the TRIzol reagent and the QIAamp viral RNA minikit (Qiagen).

Furthermore, the cDNA was synthesized through the cDNA synthesis system kit (Roche), as described by the manufacturer. The library was constructed using the Illumina Nextera DNA library preparation kit, with 100-bp paired-end reads. Sequencing of the library was performed on the HiSeq 2500 platform (Illumina).

The sequencing generated 47,628,660 reads. These were filtered, removing the adapters, sequences with a Phred quality score below 20, and reads with size less than 50 bp using Trim Galore 0.4.4 and Cutadapt (3). The rRNA reads were removed with the software SortMeRNA v2.1 (4). *De novo* assembly was performed using Trinity v2.8.4 (5), and the contigs were annotated with the Blast2GO (6) tool and aligned against the fungal nonredundant protein database and InterPro database.

A sequence 5,530 nucleotides (nt) long containing a partial viral polymerase gene was obtained from the assembly of the transcriptome of the fungus *M. irregularis* C3B. This sequence contains a predicted RNA polymerase domain specific to a negative-strand RNA virus (Prosite entry PS50525) and present at 30.19% identity (score, 473; query cover, 66%; E value, 3e−132) to the polymerase sequence of Beihai sesarmid crab virus 5 (GenBank accession number KX884794), a crustacean-associated virus, according to the CD-Search (7) and BLASTX analyses.

The assembly transcriptome generated 30,195 contigs (*N*_50_, 11,037 nt; GC content, 38.13%), of which 21,313 presented a BLAST hit result. The gene ontology (GO) distribution of the sequences for molecular function, biological processes, and cellular components is presented in Table 1.

**TABLE 1:** Top 10 gene ontology distribution of the *Mucor irregularis* C3B transcriptome

GO term level 2	No. of sequences
Biological processes	
Metabolic processes	4,817
Cellular processes	4,612
Localization	1,129
Biological regulation	1,028
Regulation of biological processes	799
Cellular component organization or biogenesis	759
Response to stimulus	658
Signaling	285
Negative regulation of biological processes	138
Positive regulation of biological processes	114
Molecular functions	
Catalytic activity	4,690
Binding	4,653
Transporter activity	531
Structural molecule activity	385
Molecular function regulator	166
Transcription regulator activity	151
Translation regulator activity	120
Antioxidant activity	25
Small-molecule sensor activity	24
Molecular transducer activity	19
Cellular components	
Membrane	2,453
Membrane part	1,882
Cell	1,852
Cell part	1,852
Organelle	1,248
Protein-containing complex	1,164
Organelle part	846
Membrane-enclosed lumen	281
Supramolecular complex	25
Extracellular region	2

### Data availability.

The partial viral sequence was deposited at GenBank under accession number MN082151. This transcriptome shotgun assembly project has been deposited at GenBank under the accession number GHMU00000000. The primary sequence data were deposited in the Sequence Read Archive under the accession number SRR8992276.
